# Primary Closure of a Partial Superior Sternal Cleft in a 27-day-old Neonate: Case Report with Short Review of Literature

**DOI:** 10.7759/cureus.4653

**Published:** 2019-05-13

**Authors:** Mithun Sundararaaja Ravikumar, Vijayanand Palanisamy, Karthik Raman, Ravi Agarwal

**Affiliations:** 1 Institute of Cardiovascular Diseases, Madras Medical Mission, Chennai, IND

**Keywords:** sternal cleft, partial, reconstruction, neonate, primary closure, subglottic hemangioma, defect, sternum, poland syndrome, pentalogy of cantrell syndrome

## Abstract

A sternal cleft is a chest wall malformation resulting from a failure of sternal fusion. It is a rare anomaly with an incidence of 2:100,000 live births representing less than a percent of all chest wall deformities. The aim of surgery is to provide bony protection over the mediastinal structures. We present a 27-day-old neonate with an upper partial sternal cleft for whom successful primary sternal closure was performed.

## Introduction

A sternal cleft (SC) is a rare chest wall malformation resulting from the failure of sternal fusion. It is a rare anomaly with an incidence of 2:100,000 live births representing less than a percent of all chest wall deformities. Surgery is mandatory to create a bony cover for the protection of mediastinal structures. We report a rare case of an upper partial sternal cleft in a 27-day-old neonate for whom successful primary sternal closure was performed.

## Case presentation

**Figure 1:**
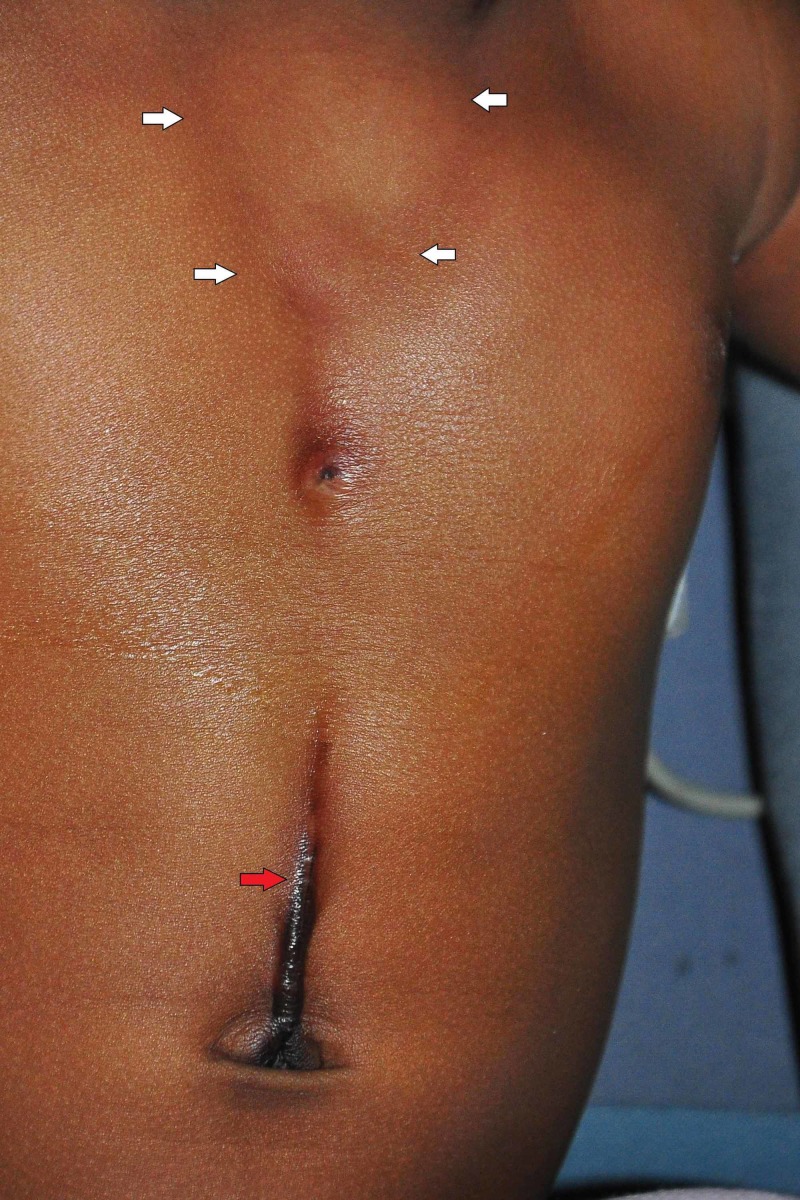
White arrows point to the margins of the sternal cleft. The red arrow points to the supraumbilical median raphe.

A 27-day-old female neonate weighing 4 kgs was referred to our department with a postnatal diagnosis of upper partial SC. The baby had an uneventful antenatal, intranatal, and perinatal period. On inspection, the baby had an intact skin cover over the chest wall (Figure 1) with a midline supraumbilical raphe.

The baby was asymptomatic except for the paradoxical movement of the upper chest wall, particularly while crying (Video 1).

**Video 1:** Video showing the paradoxical movement of the upper chest wall

In the upper part of the sternum, suprasternal and cardiac pulsations were visible through the intact skin while the lower half was fused and appeared normal. The infant was hemodynamically stable without any respiratory distress. The baby was evaluated for primary sternal closure as the presentation was early.

**Figure 2:**
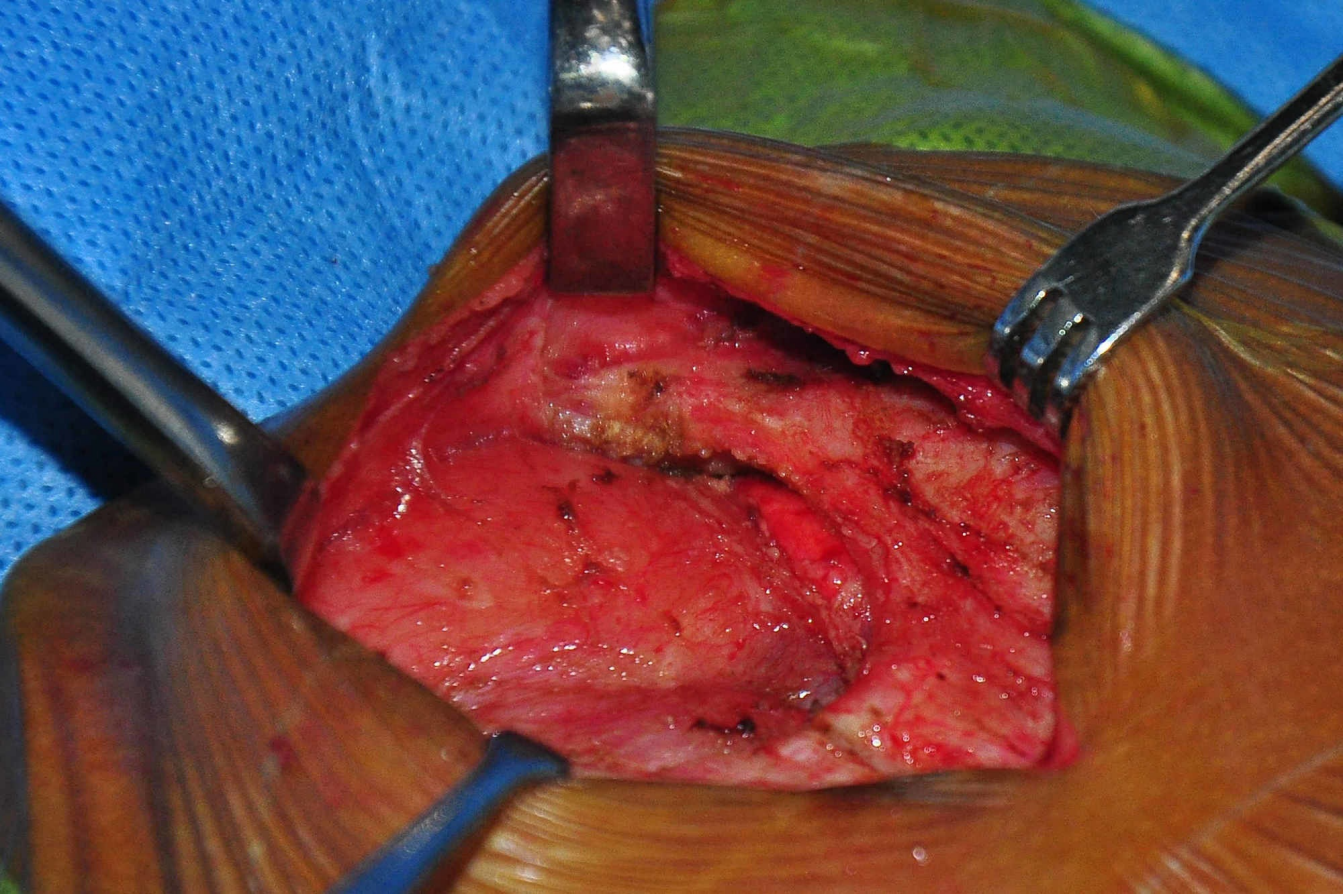
Dissected sternal cleft with exposed mediastinal structures

A midline skin incision was made and the intact lower end of the sternum was exposed. Subcutaneous tissue and the muscle plane were dissected out to expose the sternal bars on either side. The inferior surface of sternal bars was also dissected from the deep connective tissues and thymus (Figure 2).

**Figure 3:**
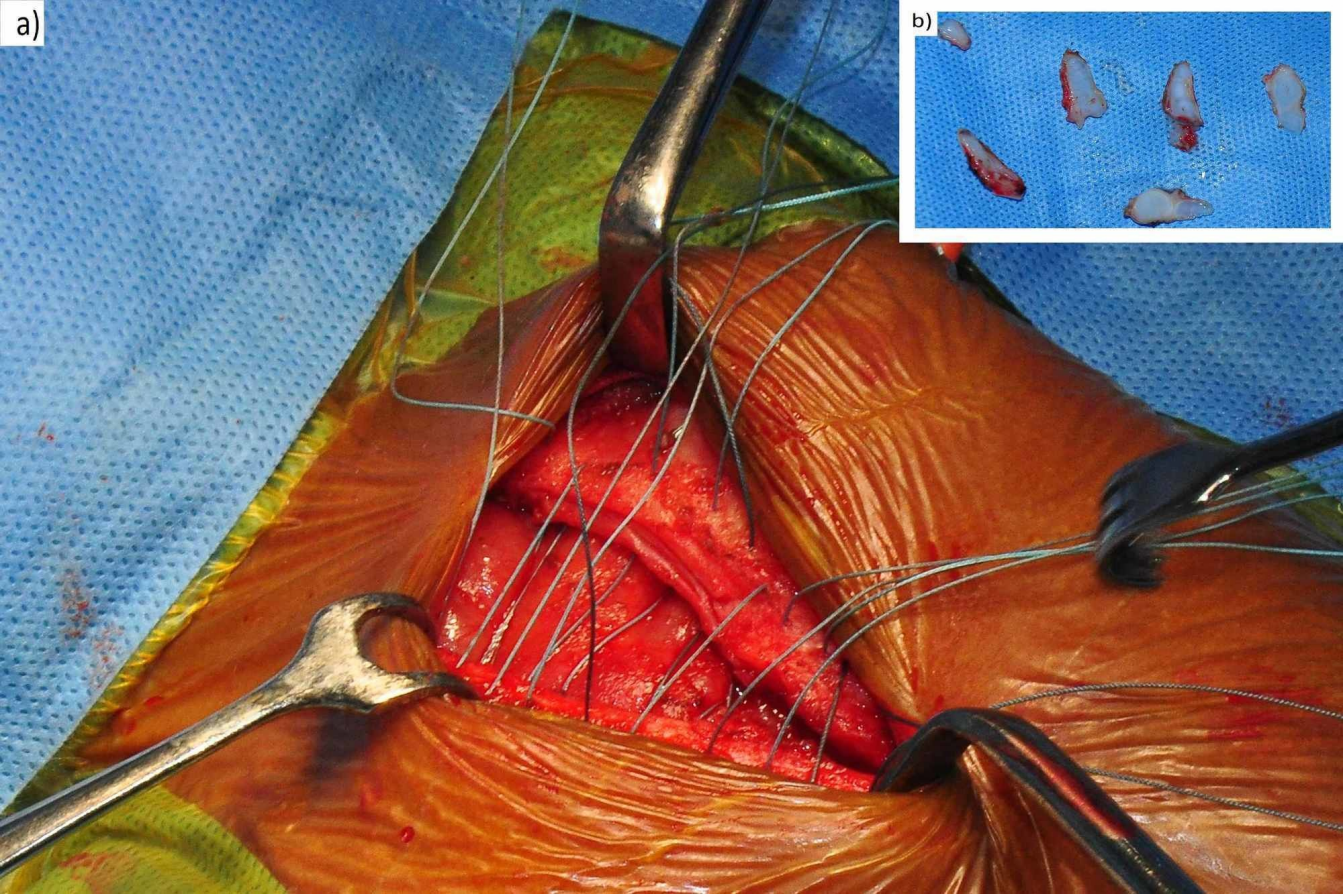
a) Sternal ends were approximated using multiple 2-0 non-absorbable sutures; b) Trimmed cartilaginous tissue

Midline sternotomy was performed in the intact sternum and the cartilaginous tissue was trimmed on both sides to create a straight margin for proper approximation. The medial portion of sternal bars was freshened by elevating the periosteum to facilitate bone healing. Prepared sternal ends were approximated using multiple 2-0 non-absorbable sutures (Figure 3).

**Figure 4:**
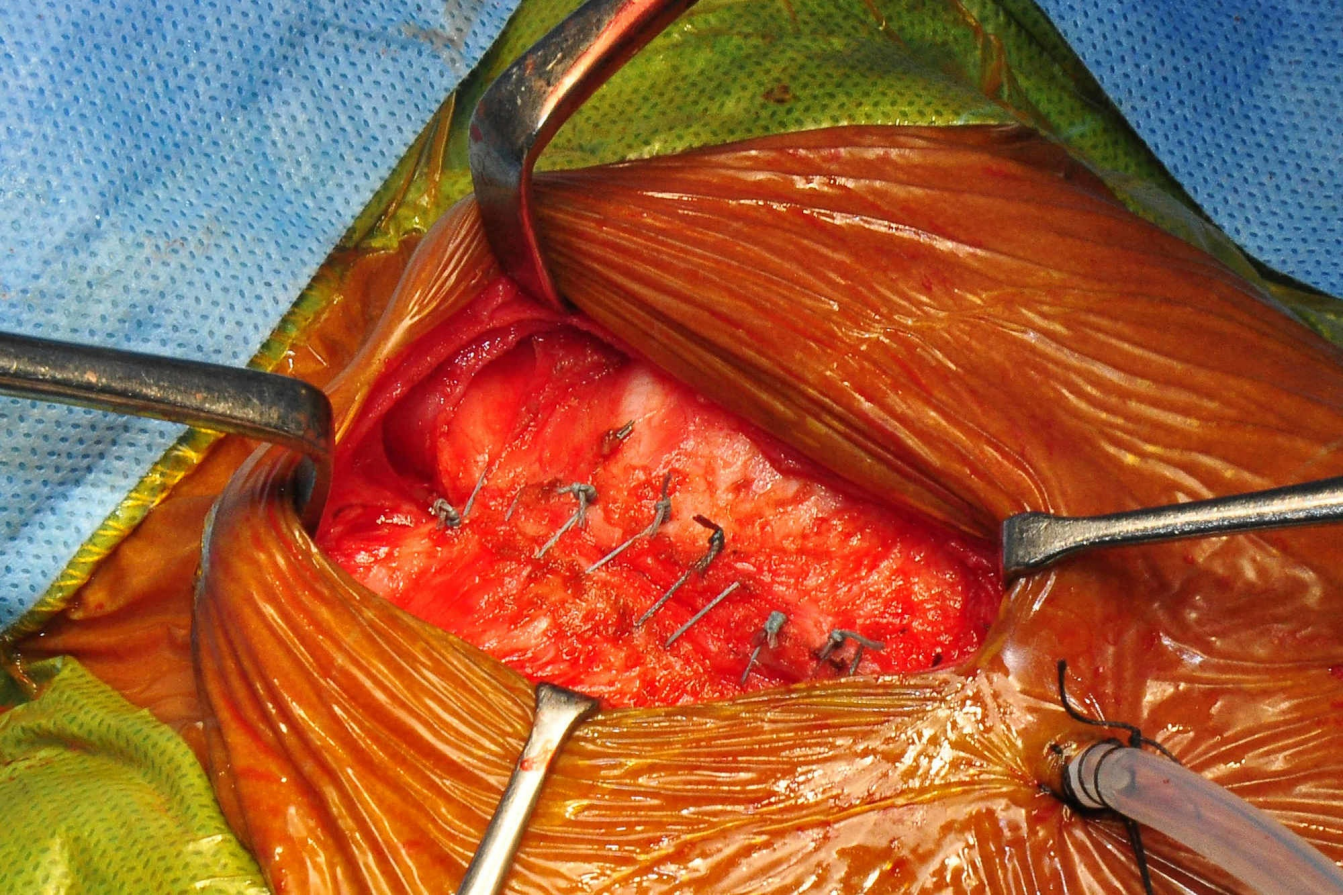
Perfectly approximated sternal ends

The baby tolerated the sternal closure well (Figure 4) without any hemodynamic instability or change in the ventilator parameters.

The baby was extubated on the day of surgery. The infant had an uneventful postoperative recovery and discharged with a stable sternum.

## Discussion

SC is a rare chest wall deformity, with very few case reports published worldwide, and corresponds to type 11 from the Willital classification of chest deformities [1]. SC is broadly classified into two groups, namely, complete and partial types. The partial SC can be further subdivided into the superior and inferior types. The most common variant is the superior partial SC constituting around two-thirds of the reported cases [2]. Hersh suggested a sternal cleft classification system consisting of four categories: (I) cleft sternum without associated anomalies, (II) cleft sternum with vascular dysplasia, (III) true ectopia cordis, and (IV) Cantrell’s pentalogy (modified Ravitch classification) [3]. Accordingly, our patient belonged to category I as per this system.

Embryologically, sternum forms from six ossifying centers. Around the end of the seventh week of gestation, the sternal bars move toward each other ventrally and gradually fuse together in a craniocaudal direction [4]. Logically, the inferior type should be the more common variant but for unknown reasons, the superior sternal cleft became the most common variant. Hox gene and maternal riboflavin deficiency were attributed to the formation of SC [5]. Further research to confirm the temporal relationship in such cases is needed before establishing etiology.

The clinical presentation of SC may vary from an incidental finding of paradoxical respiratory movements in the newborn (as in our case) during the postnatal clinical examination to severe respiratory compromise, coughing, wheezing, recurrent respiratory tract infections, and, very rarely, external cardiac compression. The paradoxical respiratory movements with the unusual movements of the chest with a depression in the skin usually give clues to diagnosing this condition. The lack of bony protection increases the susceptibility of trauma to the heart and great vessels, dyspnea, decreased lung aeration, and cough reflex, which could lead to an increase in the incidence of respiratory infections.

SC may be associated with underlying intracardiac defects and anomalies of the aortic arch. It may also be part of syndromes such as Poland syndrome and pentalogy of Cantrell syndrome [6]. Cardiac, ophthalmologic, and neurologic workup should be done before undergoing surgery. With the presence of respiratory stridor, bronchoscopy is also warranted to rule out associated subglottic hemangioma.

Burton did the first successful repair of the superior sternal cleft in 1947 and since then [7], early operative treatment of this anomaly has been advocated to ensure successful primary repair, circumvent respiratory difficulties, and gain protection for the mediastinal structures.

Surgery is the first line treatment for SC. The aim of the surgery is also to provide bony protection for the mediastinal structures and surgery should be done at the earliest. The most crucial factor determining the surgical technique is the age of the patient. In infancy, the sternum will be pliable and allows primary closure without the need for any prosthetic material, thereby avoiding complications like infection, aneurysm, and erosion of surrounding structures associated with a prosthesis.

But once age advances, sternal ossification would take place and eliminates the possibility of primary closure. Hence, in older children, sliding chondroplasty with or without dislocation of the sternoclavicular junction or autologous bone grafts harvested from the rib, iliac crest, calvaria, and tibia are required to achieve the bony cover for mediastinal structures.

Some of the other popular techniques include cartilage resection (Sally technique), sliding chondrotomy (Sabiston technique), use of muscle flaps, cartilage graft bridging, and prosthetic closure using titanium plates. The Sally technique [8] incorporates the resection of cartilaginous xiphoid and approximation of both sternal halves. Sabiston [9] described a sliding chondrotomy technique in which oblique incisions through the costal cartilage are made to increase the length of the cartilage. Cartilage graft bridging is a technique by which the gap of the sternal cleft is bridged using costal cartilage.

During primary repair, it is critically important to monitor the hemodynamic and respiratory parameters especially during sternal approximation and in the immediate perioperative period as primary sternal approximation might lead to the compression of the underlying structures. The complication rate of surgery is generally low and mortality is rare.

Good intensive team support with a cardiopulmonary bypass as backup is necessary to manage postoperative respiratory distress. The pediatric anesthetist should be aware of the possible risks of cardiac, lung, great vessel, and nerve injuries.

## Conclusions

A sternal cleft is a rare condition but early diagnosis and surgical intervention during early infancy will give a good result with a very low complication rate. Surgery in the form of either primary closure or sliding chondroplasty with or without dislocation of the sternoclavicular junction should be decided based on age at the time of presentation. A multidisciplinary team is mandatory in managing a patient with a sternal cleft for a successful outcome.
